# The biological activity of bispecific trastuzumab/pertuzumab plant biosimilars may be drastically boosted by disulfiram increasing formaldehyde accumulation in cancer cells

**DOI:** 10.1038/s41598-019-52507-9

**Published:** 2019-11-07

**Authors:** Tatiana V. Komarova, Ekaterina V. Sheshukova, Ekaterina N. Kosobokova, Vyacheslav S. Kosorukov, Anastasia V. Shindyapina, Fedor A. Lipskerov, Polina S. Shpudeiko, Tatiana E. Byalik, Yuri L. Dorokhov

**Affiliations:** 1 Vavilov Institute of General Genetics, Russian Academy of Sciences, 119991 Moscow, Russia; 2 A.N. Belozersky Institute of Physico-Chemical Biology, Lomonosov Moscow State University, 119991 Moscow, Russia; 3 N.N. Blokhin National Medical Research Center of Oncology, Ministry of Health of the Russian Federation, 115478 Moscow, Russia; 4 Faculty of Bioengineering and Bioinformatics, Lomonosov Moscow State University, 119991 Moscow, Russia; 5 I.M.Sechenov First Moscow State Medical University, 119991 Moscow, Russia; 6 Present Address: Brigham and Women’s Hospital, Harvard Medical School 77 Avenue Louis Pasteur, Boston, MA 02115 USA

**Keywords:** Applied immunology, Breast cancer

## Abstract

Studies of breast cancer therapy have examined the improvement of bispecific trastuzumab/pertuzumab antibodies interacting simultaneously with two different epitopes of the human epidermal growth factor receptor 2 (HER2). Here, we describe the creation and production of plant-made bispecific antibodies based on trastuzumab and pertuzumab plant biosimilars (bi-TPB-PPB). Using surface plasmon resonance analysis of bi-TPB-PPB antibodies binding with the HER2 extracellular domain, we showed that the obtained Kd values were within the limits accepted for modified trastuzumab and pertuzumab. Despite the ability of bi-TPB-PPB antibodies to bind to Fcγ receptor IIIa and HER2 oncoprotein on the cell surface, a proliferation inhibition assay did not reveal any effect until α1,3-fucose and β1,2-xylose in the Asn297-linked glycan were removed. Another approach to activating bi-TPB-PPB may be associated with the use of disulfiram (DSF) a known aldehyde dehydrogenase 2 (ALDH2) inhibitor. We found that disulfiram is capable of killing breast cancer cells with simultaneous formaldehyde accumulation. Furthermore, we investigated the capacity of DSF to act as an adjuvant for bi-TPB-PPB antibodies. Although the content of ALDH2 mRNA was decreased after BT-474 cell treatment with antibodies, we only observed cell proliferation inhibiting activity of bi-TPB-PPB in the presence of disulfiram. We concluded that disulfiram can serve as a booster and adjuvant for anticancer immunotherapy.

## Introduction

Breast cancer is the most commonly occurring cancer in women and the second most common cancer overall. Each year, breast cancer kills more than 500,000 women worldwide. Approximately 20–30% of patients with breast cancer, the so-called HER2-positive and most dangerous form of cancer, show overproduction of the human epidermal growth factor receptor 2 (HER2). The *HER2* gene maps to chromosome 17q21 and encodes a 1,255 amino acid, transmembrane glycoprotein tyrosine protein kinase, ErbB2, with a mass of 185 kDa^1^.

Abnormal activity of HER2 causes accelerated metastasis and resistance to therapies^2^. Success in treating HER2+ breast cancer is associated with the introduction of trastuzumab into medical practice, which is based on humanized monoclonal antibodies produced by mouse hybridomas^3^. An antibody injected into the patient’s bloodstream interacts with the extracellular part of HER2 and inhibits the division of cancer cells but rarely causes the death of cancer cells. In combination with chemotherapy, trastuzumab antibodies have a pronounced therapeutic effect, reduce the risk of developing distant metastases and increase the life expectancy of patients^4^.

Trastuzumab is currently used as a first-line drug for treating breast cancer, but its effect is limited in treating metastatic breast cancer with low HER2 expression.

In addition, when treating breast cancer with trastuzumab, the incidence of resistant cellular forms is high^5–7^. One way to overcome this problem is to use an antibody capable of recognizing another domain of the extracellular part of HER2 that is different from the trastuzumab recognition site^8^. Trastuzumab interacts with the IV subdomain (amino acids 480 to 620), while pertuzumab, which has recently entered clinical practice, interacts with the II subdomain of dimerization (amino acids 165 to 310), blocking the dimerization of HER2 and HER3^9^. Because pertuzumab and trastuzumab block HER2 in different domains, the combination of these antibodies is more effective than individual antibodies because their mechanisms of action complement each other, providing a synergistic effect^10^ - a stronger blockade of HER2-positive tumour cell proliferation and the ability to treat forms of cancer resistant to trastuzumab^8,11^. The use of pertuzumab in combination with trastuzumab and docetaxel chemotherapy has improved clinical outcomes, justifying the use of this approach^12^.

Further improvements in breast cancer therapy are associated with bispecific antibodies^13^. In general, bispecific antibodies interact simultaneously with two different epitopes located on the same antigen or on two different antigens. Notably, (a) although in some cases bispecific antibodies do not provide a functional advantage over a combination of two corresponding monospecific antibodies, they often become economically advantageous because they do not require two separate production processes^14^ and (b) are an effective tool for finding new mechanisms of influence on cancer^15^.

Various approaches have been developed to obtain bispecific antibodies, which ultimately made it possible to solve problems related to their stability and solubility. During the progress of this research, it became apparent that there is no universal design for generating bispecific antibodies. For each particular case, it was necessary to develop its most acceptable design^13^.

Using trastuzumab and pertuzumab, bispecific antibodies that retain the ability to bind HER2 and exhibit pharmacokinetic properties similar to the usual immunoglobulin G molecule were also obtained^16^. Moreover, an afucosylated bispecific anti-HER2 antibody, MBS301, has recently been created based on trastuzumab and pertuzumab, which preserves the synergistic effect of the combined use of trastuzumab and pertuzumab and acquires the enhancement of antibody-dependent cellular cytotoxicity (ADCC) via glycoengineering of the Fc N-linked glycan^17^.

Trastuzumab and pertuzumab used in clinical practice are obtained in Chinese hamster ovary cell culture. However, plant cells represent an alternative system for obtaining trastuzumab and pertuzumab biosimilars because the plant cell has mechanisms of protein synthesis and posttranslational modification (glycosylation, phosphorylation) similar to that of an animal cell. In general, plants have additional advantages compared to mammalian cells when used in the production of pharmaceutical proteins as “factories”: (a) they do not contain viruses or their genetic material and prions that are pathogenic to humans, and (b) they do not require the use of expensive equipment (for example, fermenters), cultural media and maintenance of sterility. The cost of growing the plants that are usually used for recombinant protein production is incomparably lower than the cost of cultivating bacterial, yeast or animal cells^18^. The technology of “transient expression” of proteins in a plant makes it possible to accumulate foreign protein to a level reaching 10–30% of the total soluble protein in a short time (5–10 days)^19^. We used a method in which the target gene is cloned into a binary vector with its subsequent delivery to the plant cells using agroinfiltration. This approach allowed us to obtain HER2-specific trastuzumab as its plant biosimilar (TPB)^20^. Using this technique, we also produced pertuzumab plant biosimilar of (PPB) and afucosylated variants of TPB and PPB^21^.

Here, we investigated the properties of bispecific antibodies that we developed on the basis of TPB and PPB (bi-TPB-PPB). To create bi-TPB-PPB, we used two designs that allowed the production of two types of antibodies containing the variable domains of TPB and PPB. Although the two bi-TPB-PPB molecules differed from each other in their ability to bind the HER2 and Fcγ receptor IIIa (FcγRIIIa), both types of antibodies were not able to inhibit the proliferation of the HER2+ line BT-474 unless antibodies were obtained in transgenic (ΔXTFT) *Nicotiana benthamiana* plants with knock out of genes encoding β1,2-xylosyltransferase (ΔXT) and α1,3-fucosyltransferase (ΔFT). However, in the presence of disulfiram, known as an agent for the treatment of alcoholism, both types of bi-TPB-PPB inhibited proliferation of BT-474 cells and even significantly exceeded the efficiency of TPB and PPB. We concluded that disulfiram can serve as a booster and adjuvant for anticancer immunotherapy.

## Results

### Creation and expression of bispecific trastuzumab/pertuzumab plant biosimilars

**Figure 1:**
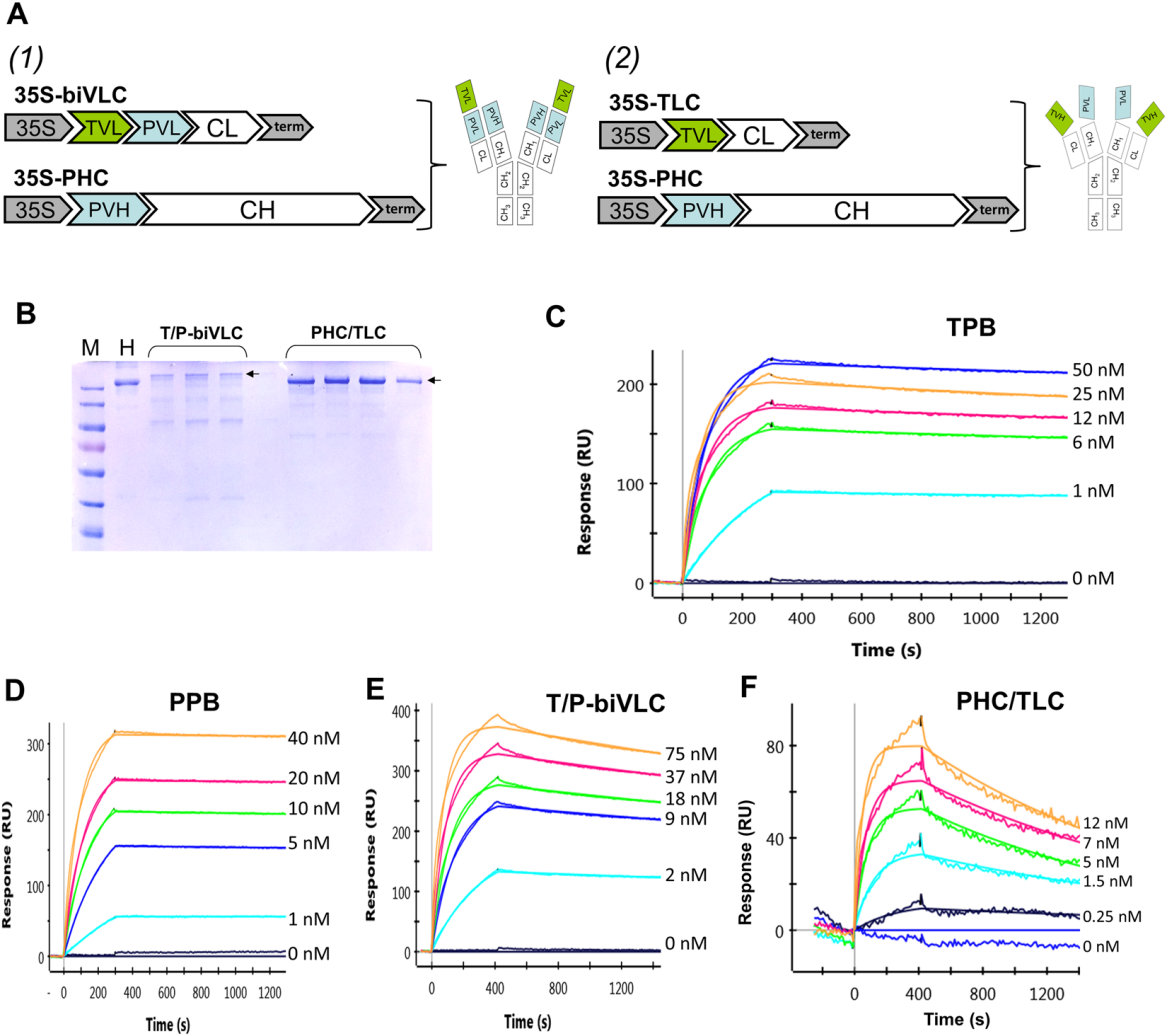
Bispecific trastuzumab/pertuzumab plant biosimilars and SPR analysis of their binding affinity to ErbB2-ECD antigen. (**A**) Schematic representation of the plant-made bispecific antibodies and binary vectors for their production in plants (1) T/P-biVLC and (2) PHC/TLC; 35S, *Cauliflower mosaic virus* 35S RNA promoter; term, CaMV 35S RNA terminator of transcription; TVL and PVL, variable domain of the trastuzumab and pertuzumab light chain, respectively; CL, constant domain of the pertuzumab light chain; PVH, variable domain of the pertuzumab heavy chain; CH, constant domain of the pertuzumab heavy chain. (**B**) T/P-biVLC and PHC/TLC purified by protein A affinity chromatography from plant leaf extracts 3 days after agroinoculation. Non-reducing conditions, 7.5% PAGE, Coomassie blue staining. The arrow indicates the fully assembled antibody. M, molecular weight markers; H, 1 μg of Herceptin. (**C–E**) Determination of the interaction constants between TPB (**C**), PPB (**D**), T/P-biVLC (**E**) or PHC/TLC (**F**) and ErbB2-ECD antigen. Typical sensory diagrams are shown. The concentrations of antibodies are indicated.

We tested different combinations of antibody chains and their variants. The first approach that we used involved the creation of a dual variable domain containing heavy chain (HC) and light chain (LC) in which the variable domains of each chain of trastuzumab were fused to the N-terminus of the corresponding pertuzumab chains via the linker TVAAPSVFI for the LC and ASTKGPSVF for the HC. The general design was based on previously developed bispecific antibodies for production in mammalian cell culture^16^. Unfortunately, the antibody based on the combination of the biVHC and biVLC did not assemble in the plant cell. We examined whether the combination of one bispecific chain (biVHC or biVLC) with one of the original pertuzumab chains could accumulate in plants. The variant consisting of the pertuzumab HC and biVLC designated the T/P-biVLC antibody (Fig. 1A-1) appeared to be applicable: the bispecific antibodies were produced in plant cells at levels reasonable for further extraction and purification. According to the bispecific antibody classification^13^, our first design of bispecific antibodies belongs to the dAb-IgG group with an appended LC variable domain consisting of the heavy chain of pertuzumab and the LC of trastuzumab.

**Figure 2:**
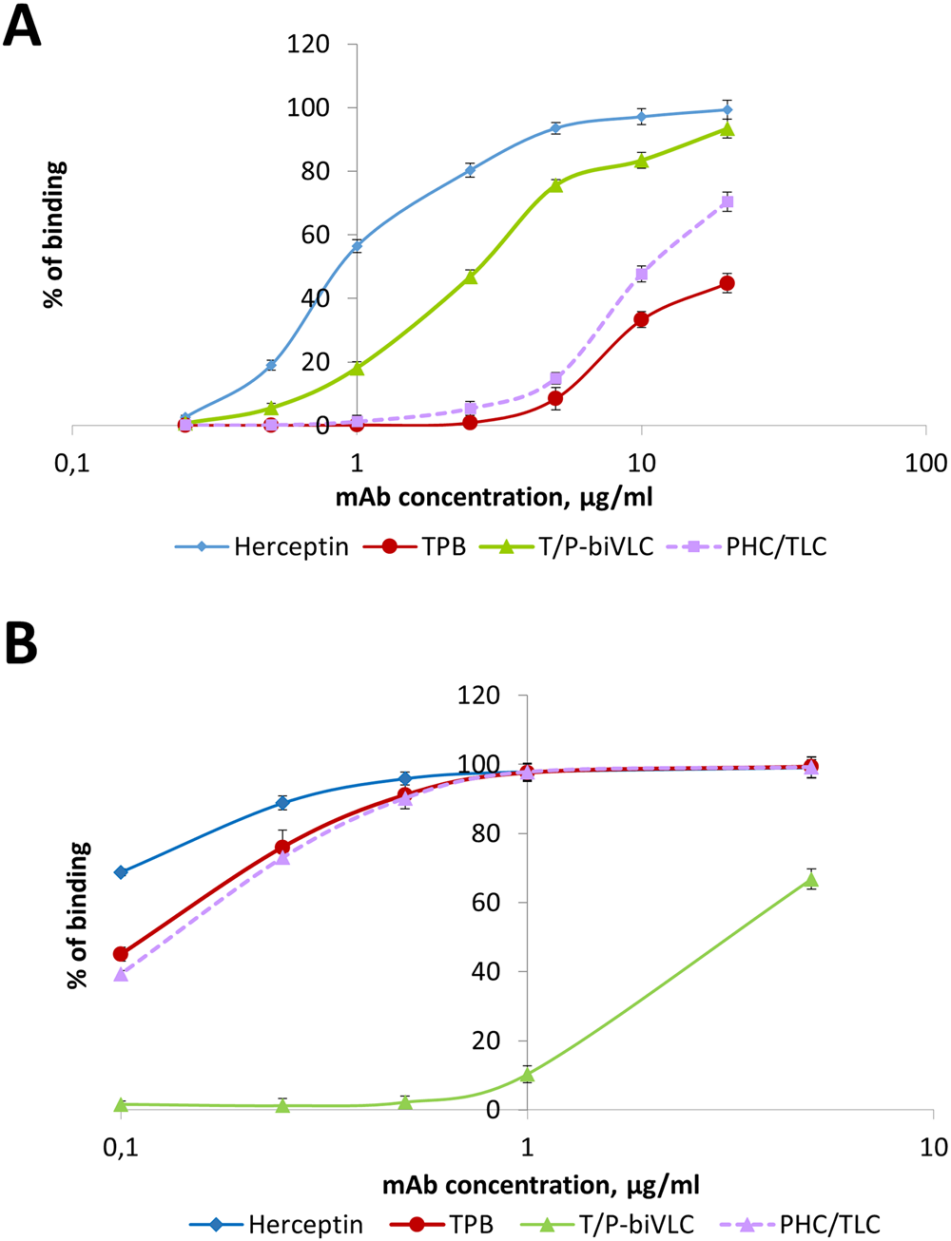
Analysis of the affinities of TPB and the bispecific antibodies T/P-biVLC and PHC/TLC to the FcγRIIIa (**A**) and HER2 oncoprotein (**B**) on the cell surface as shown by flow cytofluorimetry. (**A**) Binding of TPB, T/P-biVLC and PHC/TLC to СНО-К1.С16-cells with exposed FcγRIIIa on the surface. Herceptin is used as control. Cells were incubated with antibodies at concentrations from 0.25 to 20 µg/ml. The ordinate axis corresponds to the percentage of cells bound to antibodies as indicated by fluorescein isothyocyanate (FITC) fluorescence. The mean values with standard errors are indicated. (**B**) Binding of TPB, T/P-biVLC and PHC/TLC to HER2 oncoprotein on the surface of BT-474 cells. Herceptin is used as control. Cells were incubated with antibodies at concentrations from 0.1 to 20 µg/ml. The ordinate axis corresponds to the percentage of cells bound to antibodies as indicated by FITC fluorescence. The mean values with standard errors are indicated.

Another approach we used was the combination of the LC from one antibody and the HC from the other. Two resulting variants were THC/PLC and PHC/TLC containing the trastuzumab HC (THC) with the pertuzumab LC (PLC) or vice versa. Both antibodies accumulated in plant cells at comparable levels. The design of these antibodies represents an example of two-in-one symmetric bispecific antibodies^13^. After the preliminary functional tests, we chose PHC/TLC (Fig. 1A-2) for further study.

Thus, for the further study, we selected two variants of bi-TPB-PPB antibodies, both of which involve the use of a symmetrical design that allowed us not to exploit the popular knob-into-hole approach^22^, since both assemblies used the pertuzumab HC, rather than a combination of two heterologous HC, i.e., two disulphide bonds between the C_H_2 domains of HC ensured the stability of the assembled antibody.

We analysed by electrophoresis in 7.5% polyacrylamide gel (PAAG) in non-reducing conditions both types of bi-TPB-PPB accumulated in *N. benthamiana* leaves 3 days after agroinoculation and purification by protein A affinity chromatography. Figure 1B shows that the level of antibody accumulation in plants is sufficient for further study. Mass spectroscopic analysis of the peptides of the bi-TPB-PPB antibodies confirmed the amino acid composition of the antibodies isolated from the leaves (data not shown).

### Determination of the parameters of the affinity properties of bi-TPB-PPB using the surface plasmon resonance (SPR) method

After purification on an affinity column, we analysed the bi-TPB-PPB antibodies using the SPR approach (ProteOn SPR System, BioRad). We evaluated, in comparison with Herceptin (Hoffmann-La Roche), Perjeta (Hoffmann-La Roche), TPB (Fig. 1C) and PPB (Fig. 1D), the affinity of bi-TPB-PPB (Fig. 1E,F) binding to the extracellular domain (ECD) of the HER2 protein.

As shown in Table 1, the K_D_ value of PHC/TLC was determined to be 1.81 ± 0.76 × 10^−10^ M, which roughly corresponds to Herceptin (1.73 ± 0.95 × 10^−10^ M) but was worse than Perjeta (0.329 ± 0.24 × 10^−10^ M), TPB (0.17 ± 0.02 × 10^−10^ M) and PPB (0.39 ± 0.14 × 10^−10^ M). The K_D_ value of T/P-biVLC was determined to be 0.50 ± 0.10 × 10^−10^ M, which roughly corresponds to Perjeta and PPB but was superior to that of PHC/TLC and Herceptin.

**Table 1:** The affinities of anti-HER2 bi-TPB-PPB antibodies binding to human HER2-ECD.

Antibody	K_a_ (10^5^ M^−1^ s^−1^)	K_d_ (10^−5^ s^−1^)	K_D_ (10^−10^ M)
Herceptin	5.9 ± 1.8	6.2 ± 1.5	1.73 ± 0.95
Perjeta	16.4 ± 10.6	3.7 ± 1.38	0.329 ± 0.24
TPB	21.0 ± 11.0	5.7 ± 1.1	0.17 ± 0.02
PPB	12.2 ± 8.1	4.0 ± 2.1	0.39 ± 0.14
PHC-TLC	33.4 ± 16.0	50.2 ± 7.47	1.81 ± 0.76
T/P-biVLC	18.7 ± 1.1	9.11 ± 1.6	0.50 ± 0.10

Experimental error is the SD from three independent determinations.

In general, the obtained affinity indicators are within the limits adopted for modified trastuzumab (Kd = 10^−8^-10^−10^ M)^23,24^ and pertuzumab^16^.

### Evaluation of the binding of bi-TPB-PPB antibodies to FcγRIIIa and HER2 on the cell surface

Using flow cytofluorometry, the ability of bi-TPB-PPB antibodies to interact with the FcγRIIIa was evaluated. For this, the cell line CHO-K1.Cl6 (ECACC 15042901) carrying FcγRIIIa on the surface of cells was used. Figure 2A shows that the T/P-biVLC variant had a more pronounced ability to interact with FcγRIIIa than PHC/TLC, which seems somewhat unexpected because both bi-TPB-PPB antibodies have identical pertuzumab heavy chains, and the plant biosimilar, PPB, was able to bind to CHO-K1.Cl6 cells comparable to Perjeta (data not shown).

When studying the binding of bi-TPB-PPB antibodies to the HER2 oncoprotein on the surface of BT-474 cells, we found that there was almost no difference between TPB and PHC/TLC, while the T/P-biVLC variant was noticeably inferior to them (Fig. 2B).

### Fucose elimination from bi-TPB-PPB antibodies N-linked glycan allows them to inhibit BT-474 cell proliferation

**Figure 3:**
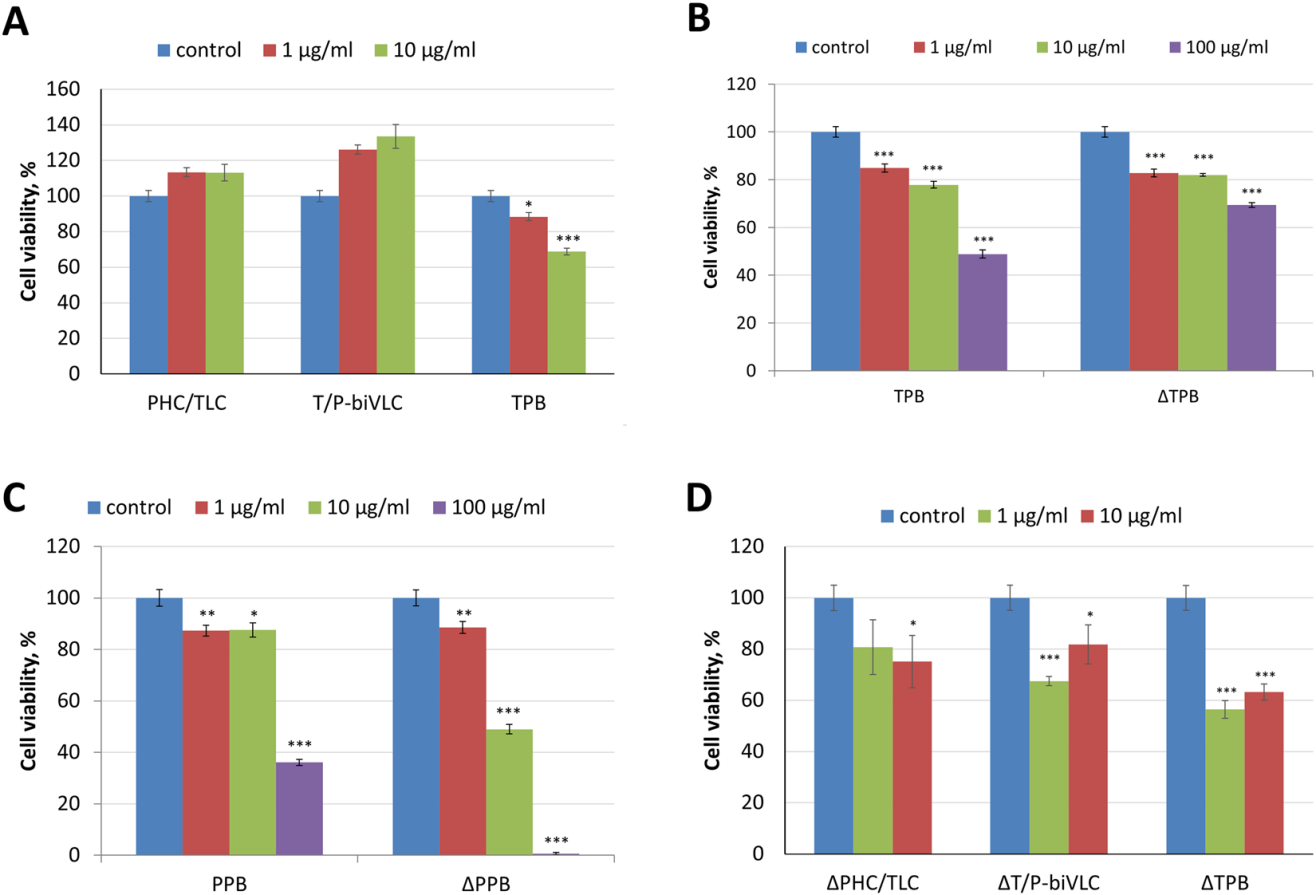
Glycomodification of bispecific antibodies increases their activity. (**A**) Effects of PHC/TLC, T/P-biVLC and TPB antibodies on growth of the breast cancer cell line BT-474 in MTT assays. Proliferation inhibition effect of the indicated concentrations (1 or 10 μg/ml) of each antibody. Control, cells treated with rituximab (Hoffmann-La Roche) in the corresponding concentrations (1 or 10 μg/ml). *p < 0.05; ***p < 0.001 (Mann-Whitney test) for the difference compared to the control. (**B**) Inhibition of proliferation of BT-474 cells by TPB and TPB-ΔXTFT (ΔTPB) according to the results of the MTT test. Control, cells not treated with antibodies. The mean values and standard error are given. ***p < 0.001 (Mann-Whitney test) for statistical significance of the difference compared to the control. (**C**) Inhibition of proliferation of BT-474 cells by PPB and PPB-ΔXTFT (ΔPPB) according to the results of the MTT test. Control, cells incubated without antibodies. The mean values and standard errors are given. *p < 0.05; **p < 0.01; ***p < 0.001 (Mann-Whitney test) for statistical significance of the difference compared to the control. (**D**) Effects of bispecific antibodies with eliminated fucose and xylose residues from N-linked glycan on the growth of the BT-474 cell line in the MTT assay. Proliferation inhibition effect of 1 or 10 μg/ml of the glycomodified antibodies ΔPHC/TLC and ΔT/P-biVLC compared to ΔTPB. Control, cells treated with rituximab (Hoffmann-La Roche) 1 or 10 μg/ml. *p < 0.05; ***p < 0.001 (Mann-Whitney test) for statistical significance of the difference compared to the control.

Despite the ability of bi-TPB-PPB antibodies to bind HER2 oncoprotein on the surface of cancer cells, Fig. 3 shows that the proliferation inhibition assay did not reveal any inhibition of BT-474 cell proliferation in the presence of both types of bi-TPB-PPB antibodies. In contrast, TPB (Fig. 3B) and PPB (Fig. 3C) and their glycomodified variants obtained in transgenic (ΔXTFT) *N. benthamiana* plants with knockout of the genes encoding β1,2-xylosyltransferase (ΔXT) and α1,3-fucosyltransferase (ΔFT) (TPB-ΔXTFT and PPB-ΔXTFT) effectively inhibited the proliferation of BT-474 cells. Moreover, the effectiveness of PPB-ΔXTFT was markedly increased compared to its unmodified version.

We assumed that because our bi-TPB-PPB antibodies have a PPB heavy chain, bi-TPB-PPB antibodies accumulated in transgenic *N. benthamiana* ΔXTFT plants will have a clear ability to inhibit the proliferation of cancer cells. Indeed, as Fig. 3D shows and according to our expectations, glycomodified bi-TPB-PPB antibodies of both types (ΔPHC/TLC and ΔT/P-biVLC) could inhibit the proliferation of BT-474 cells.

### Analysis of the *ALDH2* mRNA content in BT-474 cells treated with bi-TPB-PPB antibodies

**Figure 4:**
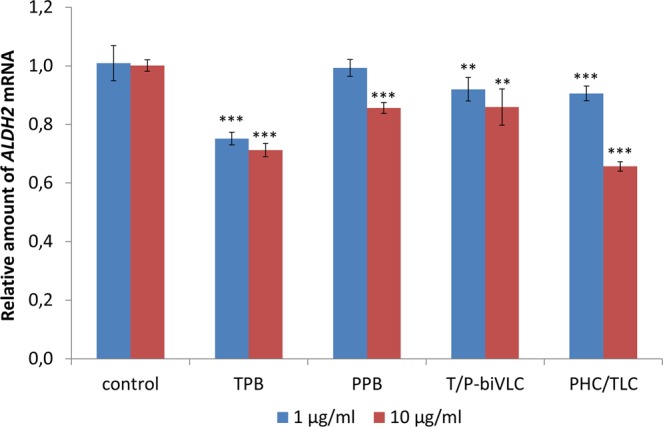
The relative content of *ALDH2* mRNA in BT-474 cells treated with HER2-specific monoclonal antibodies as shown by qRT-PCR analysis. The effect of 1 or 10 μg/ml of TPB, PPB, PHC/TLC or T/P-biVLC antibodies compared to the control – cells treated with 1 or 10 μg/ml rituximab (Hoffmann-La Roche). Cells were incubated with antibodies for 5 days. The mean values and standard error are given. **p < 0.01; ***p < 0.001 (Mann-Whitney test) for statistical significance of the difference compared to the control.

The level of *ALDH* mRNA in HER2-positive breast cancer cells was shown to be much higher than that of HER2-negative cells^25,26^. However, trastuzumab-mediated inhibition of proliferation of BT-474 cells was accompanied by suppression of the expression of the *ALDH1* and* ALDH2* genes^27^. To test the response of BT-474 cells to bi-TPB-PPB antibodies, we analysed the content of *ALDH2* mRNA as a possible response to antibody exposure. Figure 4 shows that the *ALDH2* mRNA content in BT-474 cells treated with bi-TPB-PPB antibodies of both types, as well as TPB and PPB antibodies, was sharply reduced. This finding suggests that although after exposure to bi-TPB-PPB antibodies, we did not observe inhibition of BT-474 cell proliferation, at the transcriptome level, these cells respond to bi-TPB-PPB antibodies.

### Disulfiram (DSF) inhibits the proliferation of cancer cells and stimulates the intracellular accumulation of endogenous formaldehyde

**Figure 5:**
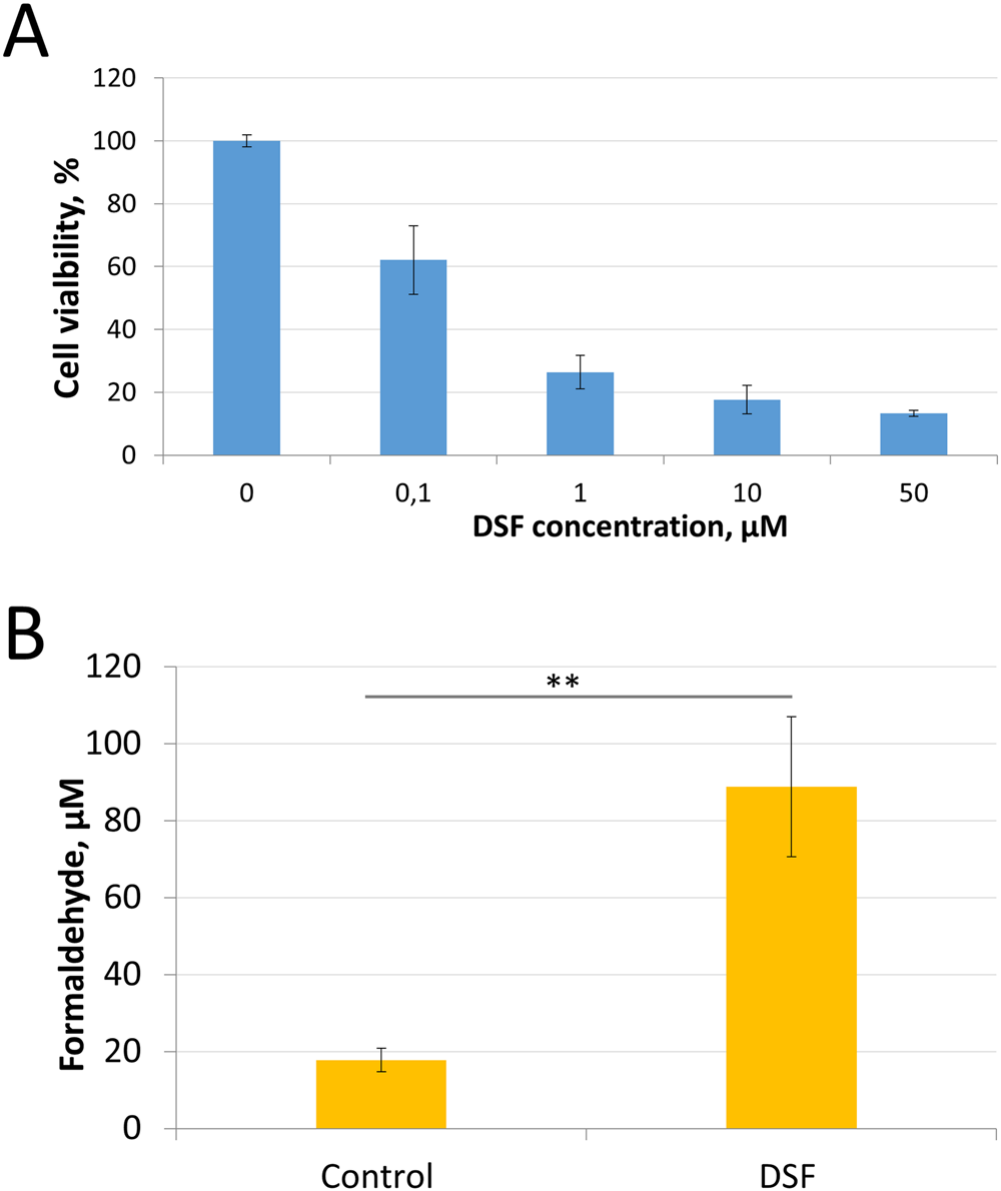
Assessment of the disulfiram effects on the proliferation of cancer cells *in vitro* and the accumulation of endogenous formaldehyde in them. (**A**) - Inhibition of proliferation of BT-474 cells by different concentrations of disulfiram according to the results of the MTT test. Control, cells incubated with the corresponding amount of vehicle (DMSO). The mean values and standard errors are given. p < 0.001 (Mann-Whitney test) for statistical significance of the difference compared to the control. (**B**) – The content of intracellular endogenous formaldehyde normalized to total protein content in BT-474 cell lysates after DSF (1 μM) treatment. The mean values and standard errors are given. **p < 0.01 (Mann-Whitney test) for statistical significance of the difference compared to the control.

We hypothesized that the anticancer effect of bi-TPB-PPB antibodies will become more pronounced when cancer cells are sensitized, for example, by treatment with disulfiram (DSF), a known ALDH2 inhibitor and promising adjuvant for anticancer therapy^28^. First, we determined the cytotoxic effect of DSF on BT-474 cells. Figure 5A shows that DSF effectively inhibited cancer cell proliferation, and its degree was proportional to the concentration in cell culture. Because the most striking and proven effect of DSF on human cells is associated with the inhibition of aldehyde dehydrogenases, an increase in the intracellular content of formaldehyde may occur^29^. Figure 5B shows that, even with a low concentration of DSF (1 μM), the intracellular formaldehyde content increased by more than 4 times.

Thus, we confirmed that DSF-induced inhibition of aldehyde dehydrogenase activity can lead to endogenous formaldehyde accumulation in cells.

### DSF drastically increases the anticancer activity of HER2-specific monoclonal antibodies

**Figure 6:**
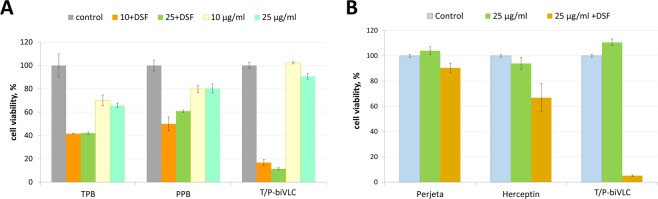
Disulfiram can serve as an adjuvant for anticancer antibodies. (**A**) Effects of disulfiram on the growth of the breast cancer cell line BT-474 in MTT assays. Proliferation inhibition effect of the indicated concentrations (10 or 25 μg/ml) of TPB, PPB or T/P-biVLC *per se* or in the presence of 0.1 μM DSF. Control, cells treated with rituximab (Hoffmann-La Roche). p < 0.01 (Mann-Whitney test) for statistical significance of the difference between the effect of TPB + DSF or PPB + DSF and T/P-biVLC + DSF. (**B**) Effects of disulfiram on growth of the trastuzumab-resistant breast cancer cell line BT-474 Clone 5 in MTT assays. Proliferation inhibition effect of 25 μg/ml Perjeta, Herceptin and T/P-biVLC *per se* or in the presence of 0.1 μM DSF. Control, cells treated with rituximab (Hoffmann-La Roche). P < 0.01 (Mann-Whitney test) for statistical significance of the difference between the effect of Perjeta + DSF or Herceptin + DSF and T/P-biVLC + DSF.

DSF will likely enhance the anticancer effect of HER2-specific monoclonal antibodies. Figure 6A shows that DSF significantly enhanced the ability of TPB and PPB to inhibit the proliferation of BT-474 cells; moreover, the observed effect is likely not to be additive but synergistic, which was especially noticeable when testing T/P-biVLC. Unexpectedly, DSF was able to sensitize even BT-474 Clone 5 cells resistant to trastuzumab (Fig. 6B), mediating the striking response of this cell line to T/P-biVLC antibodies.

We concluded that DSF can be used as an adjuvant for anticancer antibodies.

## Discussion

The interest in the creation of anticancer bispecific antibodies is due to not only the requirement for academic fundamental knowledge but also by the practical need to improve immunotherapy. To create IgG-based bispecific antibodies, an enormous range of antibody designs has been developed, including a variety of variants and combinations of both light and heavy chains and variable domains^13^. Although for each case it is necessary to select the optimal variant, the examples of successful implementation in therapeutic practice of Emicizumab (Hemlibra)^30^ and blinatumomab (Blincyto)^31^ for the treatment, respectively, of haemophilia and lymphoblastic leukaemia gives hope for the development of effective bispecific antibodies for the treatment of breast cancer^16,17,32^.

Here, to create bi-TPB-PPB antibodies, we used two designs that allowed the production of two types of antibodies containing the variable domains of TPB and PPB (Fig. 1). The SPR analysis of antibody binding towards the HER2 extracellular domain showed that the Kd was within the limits accepted for modified trastuzumab and pertuzumab (Table 1).

Despite the ability of bi-TPB-PPB antibodies to bind to HER2 and FcγRIIIa on the cell surface (Fig. 2), both types of antibodies were not able to inhibit the proliferation of the HER2-positive line BT-474. The flexibility of the hinge region of the constant part of the pertuzumab heavy chain and the ability for conformational conformity of the tertiary complex HER2-trastuzumab-pertuzumab may play an important role in the anticancer activity of bi-TPB-PPB antibodies. This view is supported by the recent results of a study of the cryo-EM structure of HER2-trastuzumab-pertuzumab at 4.36 Å resolution^33^. A comparison of the cryo-EM of this tertiary structure with the crystal structures of HER2-pertuzumab and HER2-trastuzumab complexes shows that both pertuzumab and trastuzumab can be associated with HER2 simultaneously and only with small conformational changes. Moreover, the binding of one antibody does not enhance the binding of another, which contradicts the data on the cooperative and synergistic effect of pertuzumab and trastuzumab^34^. Our results from the MTT test with glycomodified bi-TPB-PPB antibodies (Fig. 3D), as well as the properties of an afucosylated bispecific anti-HER2 antibody^17^, support the role of the heavy chain constant region conformation and flexibility for the binding and functional activity of the bispecific antibodies.

To increase the effectiveness of anticancer therapy involving bispecific antibodies, in addition to solving the steric and conformational problems of the antibody itself, the usage of adjuvants to sensitize the cancer cell as a target of antibody exposure is a reasonable and attractive strategy. The use of an adjuvant is consistent with the view that the clinical synergism of pertuzumab and trastuzumab arises not from enhanced affinity but from synergy in the inhibition of HER2-mediated signalling^33^. According to our data, disulfiram could sensitize cancer cells and thus dramatically increased the effectiveness of the anticancer effect of mono- and bispecific antibodies (Figs 5 and 6). In general, the anticancer properties of disulfiram have been known for quite some time and have been confirmed in a number of cancer cell lines and animal models^35–44^. In human tissue, disulfiram is converted into an active metabolite, diethyldithiocarbamate, (DDTC)^45,46^. DDTC irreversibly inhibits liver ALDH by competing with nicotinamide adenine dinucleotide at the cysteine residue in the active site of the enzyme. Disulfiram itself inhibits cytosolic ALDH1A1 more potently than mitochondrial ALDH2^47^. Disulfiram caused inhibition of aldehyde dehydrogenases 1 and 2, which, as we have suggested^29^, leads to the formation from endogenous sources in cancer cells of aldehydes, acetaldehyde and, in particular, formaldehyde, which is known to be two times more effective in killing cancer cells than another substrate of ALDH2, acetaldehyde^29,48,49^. Here, we have shown that disulfiram causes the accumulation of endogenous formaldehyde (Fig. 5).

The HER2-specific antibodies studied by us, suppressing the content of *ALDH2* mRNA in cancer cells (Fig. 4), probably also induce the accumulation of endogenous formaldehyde, which makes an additional contribution to their anticancer properties. The presence in the culture medium of disulfiram, generating DDTC, which irreversibly inhibits the enzymatic activity of ALDH2, only synergistically enhances the action of the antibodies studied.

However, when evaluating disulfiram sensitization of cancer cells, one should keep in mind that metabolites of disulfiram, as metal ion (Cu2+/Zn2+)-binding compounds, inhibit the proteasome and activate p53, inducing apoptosis and cell death, which explains the other mechanism of its anticancer activity^45,50^. Disulfiram was also shown to inhibit the matrix metalloproteinases (MMPs)^51^, NF-kB signalling pathway^52^, P-glycoproteins^53^ and superoxide dismutase (SOD)^54,55^. The results also showed that DSF/Cu increased apoptosis of cancer cells in a dose-dependent manner^43^. When determining the molecular target responsible for the anticancer effect of disulfiram, a DDTC and copper complex (CuET) was detected in a mouse tumour, which caused the death of cancer cells by binding to NPL4, an adapter of p97^56^.

Thus, disulfiram usage may be an example of a strategy for identifying new applications of existing well-known drugs, transferring results of laboratory studies to clinical applications^29,57^. Notably, the old ideas about the use of DDTC as an immunomodulator in the treatment of cancer and AIDS should be kept in mind^28,58,59^.

## Materials and Methods

### Genetic constructs

To create the biVLC and biVHC constructs, the sequence encoding the variable part of the trastuzumab light chain (LC) or heavy chain (HC) with the signal peptide at the N-terminus was amplified using the direct primers “SP-mAbs_NcoI_dir” and “VLC-Tras_linker_BsaI_rev” or “VHC-Tras_link_start_BsaI_rev” as reverse primers for LC or HC, respectively. Plasmids encoding the trastuzumab LC and HC, previously obtained in our laboratory^20^, were used as matrices. The obtained fragments were digested with NcoI and BsaI restriction enzymes. The sequence encoding a variable fragment of the pertuzumab LC or HC and a part of the corresponding constant region was amplified using the “VLC-Pert_BsaI_dir”/“VLC-Pert_Acc65I_rev” pair of primers for LC and the “VHC-Pert_link_end_BsaI_dir”/“HC-const_ApaI_rev” pair of primers for HC. The plasmids pA16571 (for the LC) and pA16671 (for the HC) previously obtained in our laboratory^21^ were used as matrices. The obtained fragments were digested with BsaI-Acc65I (for LC) or BsaI-ApaI (for HC). In the next step, two obtained fragments encoding the LC were inserted into pA16571 digested with NcoI and Acc65I, resulting in a biVLC-encoding binary vector for expression in plants. Two fragments encoding the HC were cloned into pA16671 digested with NcoI and ApaI enzymes, resulting in a biVHC-encoding binary vector. The oligonucleotides used for cloning are listed in Supplementary Table 1.

### Plant growth conditions

Wild-type and ΔXTFT *Nicotiana benthamiana* plants^60^ were grown in a greenhouse in pots containing a mixture of leaf compost, humus, peat, and sand with a controlled day/night light cycle of 16/8 h at a temperature of 25/18 °C. Plants 11–12 weeks of age with 5–6 true leaves were used in the experiments.

### Agroinfiltration

*Agrobacterium tumefaciens* strain GV3101 was transformed with individual binary constructs and grown at 28 °C in LB medium supplemented with 50 mg/L rifampicin, 25 mg/L gentamycin and either 50 mg/L carbencillin or 50 mg/L kanamycin. An aliquot of *Agrobacterium* cell suspension from an overnight culture (2 mL) was diluted in 10 mM MES buffer (pH 5.5) supplemented with 10 mM MgSO_4_ to a final OD_600_ of 0.1. Agroinfiltration was performed on almost fully expanded *N. benthamiana* leaves still attached to the intact plant. A bacterial suspension was infiltrated into the leaf tissue using a 2-mL syringe, after which the plants were grown under greenhouse conditions at 22 °C with 16 hours of light.

### Antibody extraction and purification

Total soluble protein was extracted from agroinoculated *N. benthamiana* leaves with 10 mM sodium phosphate buffer pH 7.0. Plant extracts were analysed by electrophoresis in the absence of β-mercaptoethanol in 7.5% PAAG followed by Coomassie staining as described earlier^21^. Antibody isolation from crude plant extract was performed by affinity chromatography on Protein A Sepharose 4 Fast Flow (GE Healthcare) according to the manufacturer’s protocol. Briefly, plant material was ground in the presence of 4–5 volumes of 10 mM sodium phosphate buffer pH 7.0, followed by filtration through Miracloth and centrifugation at 10,000 *g* for 15 min. The cleared extract was loaded on the Protein A Sepharose column equilibrated with 10 mM sodium phosphate buffer pH 7.0. The extract passed 2–4 times through Sepharose to improve binding. In the next step, the column was washed once with phosphate-buffered saline (PBS) and then with 10 mM sodium phosphate buffer, pH 7.0. Elution was carried out with 100 mM glycine, pH 3.0, followed by immediate neutralization with 0.4 M Tris, pH 8.8. To estimate antibody yield in plant extracts, we used a “Total IgG ELISA-BEST” kit (Vector-Best) according to the manufacturer’s instructions. The concentration of the affinity chromatography-purified antibodies was determined using a BioDrop spectrophotometer and a BCA Protein Assay Kit (Pierce).

### Surface plasmon resonance

A study of the mono- and bispecific antibody affinity by the SPR method was performed using a ProteOn XPR36 Protein Interaction Array System (BioRad) instrument and a GLM Sensor microchip (BioRad) as previously described^61^. The antigen, which is the extracellular part of ErbB2 [ErbB2-ECD (SinoBiological)], at a concentration of 10 μg/ml was immobilized for 5 minutes at a speed of 25 μl/min on the chip surface using the amine coupling method. Antibodies were diluted to the required concentration in the working buffer (10 mM NaH_2_PO_4_/0.4 M Na_2_HPO_4_ = 10/1 with 0.05% Tween 20). The binding constants were counted according to Langmuir. Only data with χ2 < 5 for 3–9 technical repetitions were taken into account.

### Cell cultures

The BT-474 (ATCC HTB-20) cell line and trastuzumab-resistant BT-474 Clone 5 (ATCC CRL-3247) cells were obtained from the American Type Culture Collection. These cell lines were cultured in DMEM medium (PanEco) without phenol red and containing 10% foetal bovine serum (HyClone). Both cell lines feature elevated HER2 expression. The CHO-K1.CI6 cell line (ECACC 15042901) was obtained from the European Collection of Authenticated Cell Cultures and is a clone of CHO-K1 cells expressing human FcγRIIIa of the 158 V allotype anchored to glycosyl phosphatidylinositol. This cell line was cultured in DMEM medium (PanEco) with phenol red and 10% foetal bovine serum (HyClone).

### Flow cytometry

The affinity of plant-produced mono- and bispecific antibodies to HER2 antigen on the surface of the BT-474 cells and FcγRIIIa on CHO-K1.C16 cells was assessed using flow cytometry as described earlier^21^.

### Cell proliferation assay (MTT)

The effect of anti-HER-2 mAbs and disulfiram on proliferation of the human mammary adenocarcinoma cell line BT-474 and trastuzumab-resistant BT-474 Clone 5 was studied by MTT [3-(4,5-dimethylthiazol-2-yl)-2,5-iphenyltetrazolium bromide] assays^20^ with modifications. Briefly, cells (5 × 10^4^ cells per well) were seeded in 48-well plates. Twenty-four hours later, the studied substances (mAbs, disulfiram) were added. After exposure to the different drugs for 120 h, 10 µL of MTT solution (5 mg/mL in PBS) was added to each well, and the plates were incubated for 4 h at 37 °C. The MTT solution in the medium was removed by aspiration. To achieve solubilization of the formazan crystal formed in viable cells, 150 mL of dimethylsulfoxide (DMSO) was added to each well, followed by incubation for 10 minutes at 37 °C. The absorbance (A) at 590 nm and 670 nm was measured. Cell survival was calculated as the ratio of (A_590_-A_670_) in wells containing a studied substance compared to that in control wells.

### Quantitative real-time PCR analysis

Total RNA was extracted from BT-474 cells treated with antibodies using TriReagent (MRC) according to the manufacturer’s protocol. RNA concentrations were determined using a BioDrop spectrophotometer. All RNA samples had a 260/280 absorbance ratio between 1.9 and 2.1, and cDNA synthesis and qRT-PCR were performed as described earlier^62^. Target gene mRNA levels were calculated according to the equation: E_target_^ΔCPtarget (sample-reference)^^63^. PCR efficiency (E) was calculated according to the equation E = 10^(−1/slope)^ based on the standard curves. *ALDH2* gene mRNA levels were corrected to the reference gene encoding ribosomal protein L32 (*RPL32*). The oligonucleotides used for qRT-PCR are listed in Supplementary Table 1.

### Formaldehyde measurement in cell lysates

#### Cell lysate preparation

BT-474 cells were seeded in 6-well plates in 2 mL, 150,000 cells/per well. Twenty-four hours later, disulfiram (Sigma) diluted in DMSO was added up to 1 µM, and the corresponding amount of DMSO was added to the control wells. After 72 h incubation, the cells were washed with PBS and collected with 700 µL of 0.05% trypsin solution containing 0.53 mM EDTA and Hanks’ salts (PanEco) and washed twice with PBS, pH 7.4. Then, the cells were resuspended in 130 µL of PBS and frozen in liquid nitrogen. After two cycles of thawing at room temperature/freezing at −20 °C, cell lysates were cleared with centrifugation at 10000 g for 10 minutes.

#### Formaldehyde measurements

For formaldehyde concentration measurements in lysates, a protocol based on formaldehyde derivatization with Purpald reagent (Sigma) was developed. 100 µL of Purpald solution (34 mМ Purpald, 2 М NaOH) was added to 100 µL of each sample and mixed thoroughly. After 30 min incubation at room temperature, 100 µL of water was added to the mixture, and the absorbance of the solution was measured at 550 nm. The concentration of formaldehyde in the samples was defined using the calibration curve. As disulfiram inhibits cell proliferation, formaldehyde concentrations in cell lysates were normalized to total protein content assessed using the BCA Protein Assay Kit (Pierce). No less than three experiments with six biological repeats were performed.

## Supplementary information

Supplementary Table 1

## Data Availability

The authors declare that all data supporting the findings of this study are available within the article and Supplementary Information.
