# Supplementary material for: The biological activity of bispecific trastuzumab/pertuzumab plant biosimilars may be drastically boosted by disulfiram increasing formaldehyde accumulation in cancer cells
Source: Sci Rep. 2019 Nov 7;9:16168. doi: 10.1038/s41598-019-52507-9 (PMC6838051; doi:10.1038/s41598-019-52507-9)
Supplement: Supplementary file 1 — Supplementary Table 1 [file 41598_2019_52507_MOESM1_ESM.pdf]

The biological activity of bispecific trastuzumab/pertuzumab plant biosimilars may be drastically boosted by disulfiram increasing formaldehyde accumulation in cancer cells

Tatiana V. Komarova[1,2,‡], Ekaterina V. Sheshukova[1,‡], Ekaterina N. Kosobokova[3], Vyacheslav S. Kosorukov[3], Anastasia V. Shindyapina[1,2,&], Fedor A. Lipskerov[1,2], Polina S. Shpudeiko[1,2], Tatiana E. Byalik[4] and Yuri L. Dorokhov[1,2*]

[1]Vavilov Institute of General Genetics Russian Academy of Sciences, 119991, Moscow, Russia

[2]A.N. Belozersky Institute of Physico-Chemical Biology, Lomonosov Moscow State University, 119991 Moscow, Russia

[3]Blokhin Cancer Research Center, 115478 Moscow, Russia

[4]I.M.Sechenov First Moscow State Medical University, 119991 Moscow, Russia

Supplementary Table 1. Oligonucleotides used for cloning and qRT-PCR.

Name	Sequence
VLC-Pert_BsaI_dir	GGTCTCAGATATACAAATGACAC
VLC-Pert_Acc65I_rev	CTATACTTTCGGACAGGGTACC
VLC-Tras_linker_BsaI_rev	CCATCTGTCTTCATCGATATGAGACC
SP-mAbs_NcoI_dir	CCATGGGATGGAGCTGTATCATC
VHC-Pert_link_end_BsaI_dir	GGTCTCAGCCCCAGTGTTTTTGAGGTACAACTTGTGGAG
HC-const_ApaI_rev	CGGCAAGCACTAAGGGCCC
VHC-Tras_link_start_BsaI_rev	CTTCTATGCTATGGACGTGGCCTCAACTAAAGGCCCTGAGACC
RPL32_dir	CATCTCCTTCTCGGCATCA
RPL32_rev	AACCCTGTTGTCAATGCCTC
ALDH2_dir	TGCTATGATGTGTTTGGAG
ALDH2_rev	TTCTTATGAGTTCTTCTGAGG
